# The Structure, Evolution, and Gene Expression Within the Caprine Leukocyte Receptor Complex

**DOI:** 10.3389/fimmu.2019.02302

**Published:** 2019-09-26

**Authors:** John C. Schwartz, Nicholas D. Sanderson, Derek M. Bickhart, Timothy P. L. Smith, John A. Hammond

**Affiliations:** ^1^The Pirbright Institute, Woking, United Kingdom; ^2^Experimental Medicine Division, Nuffield Department of Medicine, John Radcliffe Hospital, University of Oxford, Oxford, United Kingdom; ^3^Cell Wall Biology and Utilization Research, USDA-ARS, Madison, WI, United States; ^4^Meat Animal Research Center, USDA-ARS, Clay Center, NE, United States

**Keywords:** NK cells, LRC, *KIR*, *LILR*, immunoglobulin-like receptors, goat, sheep

## Abstract

The leukocyte receptor complex (LRC) encodes a large number of immunoglobulin (Ig)-like receptors involved in the immune response, particularly in modulating natural killer (NK) cell function. The killer cell Ig-like receptors (*KIR*), the leukocyte Ig-like receptors (*LILR*), and a recently described novel Ig-like receptor family are highly variable between species, which is consistent with rapid evolution driven by selection pressure from pathogens. Among the species studied to date, only simians (such as humans) and bovids (such as cattle and goats) have an expanded complement of *KIR* genes and represent an interesting model to study *KIR* evolution. Using recently improved genome assemblies and an assembly of bacterial artificial chromosomes, we describe the structure of the LRC, and the *KIR* region in particular, in goats and compare this to sheep as the assemblies allow. These species diverged from a common ancestor ~10 million years ago and from cattle ~25 million years ago. We identified conserved *KIR* genes common to both goats and sheep and confirm a partial sheep haplotype shared between the Rambouillet and Texel breeds. Goats and sheep have independently expanded two novel *KIR* subgroups, and unlike cattle or any other mammal, they do not appear to possess a functional 3DL-lineage *KIR* gene. Investigation of LRC gene expression using available transcriptomic data for various sheep and goat tissues largely confirmed putative gene annotation and revealed that a relatively conserved *caprinae*-specific *KIR* subgroup is expressed in macrophages. The *LILR* and novel Ig-like receptors were also highly expressed across a diverse range of tissues. This further step toward our understanding of the LRC receptor repertoire will help inform future studies investigating immune response variation in these species.

## Introduction

Vertebrates have evolved numerous receptor/ligand systems for monitoring and maintaining organism health. Most notable for the high degree of polymorphism and rapid evolution between species are the major histocompatibility complex (MHC) class I and class I-like molecules and their respective somatically rearranging and germline encoded receptors. As the most rapidly evolving regions of jawed vertebrate genomes, both MHC and their germline receptor complexes are typically highly polymorphic and variable in gene content. As a result, these gene complexes are often substantially different even between closely related species. One such gene complex is the leukocyte receptor complex (LRC). Within the LRC exist numerous related genes belonging to the immunoglobulin (Ig) superfamily. These include the killer cell Ig-like receptors (*KIR*) expressed by natural killer (NK) cells and a subset of CD8 T cells, and the leukocyte Ig-like receptors (*LILR*) expressed by a larger variety of lymphoid and myeloid cell types. We also recently reported the existence of an additional novel Ig-like gene family in the LRC of many mammalian species, including seven genes in cattle and a pseudogene outside the LRC in humans. Like the *KIR* and *LILR*, these genes also vary in gene content between haplotypes and between species (1). Although little is known about these novel genes, they typically encode a single long-tailed inhibitory receptor and a variable number of short-tailed receptors which potentially antagonize the function of the inhibitory form. Thus, the LRC is a dynamic, quickly evolving region of the genome that encodes multiple related receptors.

The *KIR* genes encoded within the LRC, and the unrelated killer lectin-like receptors (*KLR*) encoded within the natural killer complex (NKC) are the major cell-surface receptors which mediate NK cell function via interaction with MHC class I ligands. In all species studied to date, these gene complexes are located on different chromosomes and have variably expanded or contracted *KIR* and *KLR* content. Mice (*Mus musculus*) have a highly expanded and variable repertoire of *KLRA* genes (2, 3), yet possess only two *KIR*-like genes located outside the LRC on chromosome X with likely alternative functions than NK cell control (4, 5). In contrast, humans have a highly expanded, gene variable and polymorphic *KIR* region, yet only a single non-functional *KLRA* gene (6). An alternative strategy has been adopted by marine carnivores that have maintained single functional *KIR* and *KLRA* genes with no evidence of gene expansion (7).

Species belonging to the *Bovidae* family, such as cattle (*Bos taurus*), goats (*Capra hircus*), and sheep (*Ovis aries*), are so far unique in that they have a highly expanded repertoire of *KLR* genes within the NKC (8, 9), and are also the only non-simian species known to have an expanded complement of *KIR* genes in the LRC (10–12). Importantly, these receptors appear to be differentially expressed in NK cells depending on the MHC genotype of the animal, implying a similar regulation of NK cell function (13). A cattle *KIR* haplotype has been characterized and localized to a telomeric end of chromosome 18, and contains eight functional and 10 non-functional *KIR* genes (11). Except for *KIR2DL1* and closely related pseudogenes, the expanded and likely functional *KIR* genes in cattle have evolved from the 3DX-lineage and are therefore more closely related to *KIR3DX1* in humans. This is in contrast to humans where the functional *KIR* genes belong to the 3DL-lineage (10, 11). The goat *KIR* region, and the LRC in general, appears to be no less complex than cattle, and is disrupted by many sequence gaps and missing sequence in the previous CHIR_2.0 assembly compared to the latest ARS1 genome assembly, in which the LRC is intact on a single contig (14).

In addition to an expanded repertoire of KIR, humans also possess 11 functional LILR, which are expressed by a diverse range of cell types. At least five of these interact with MHC class I ligands, while others appear to interact with non-MHC molecules such as tetherin (15–17). Mice possess 11 paired immunoglobulin-like receptor (*PIR*) genes which are orthologous to and arranged similarly to human *LILR* (18–20). Pigs possess at least 17 *LILR* genes and gene fragments (at least six are functional) that form two distinct phylogenetic clades (1), however their ligands remain unknown. Multiple *LILR* orthologs are also known to exist in cattle (21) although they remain largely uncharacterized. Thus, like the *KIR*, the *LILR* are also highly variable between species, however little is known about their function and polymorphism outside humans.

Goats and sheep diverged from a common ancestor ~10 Mya and from cattle ~25 Mya (22). There has been no detailed investigation of the *KIR* or other LRC genes in a non-cattle species of bovid to date and there are no *KIR* mRNA accessions for goats or sheep in GenBank. To better understand the evolution of this region we describe the LRC that assembled as one contiguous sequence in the recent high-quality goat genome assembly, ARS1 (14). We also report the first sequenced and assembled partial sheep *KIR* haplotype, derived from bacterial artificial chromosomes (BACs) and compared to the LRC in both the Oar_v4 reference assembly and the recently available long-read sheep assembly, Oar_rambouillet_v1.0. The expression pattern and likely functionality of these genes was then examined using publicly available transcriptomic data from the sheep gene expression atlas (23) and the goat gene expression atlas (24, 25) produced by the FAANG consortium (26).

## Methods

### Genome Assemblies and BAC Clones

A comparison of the assembly across the LRC between the previous the goat reference assembly, CHIR_2.0, and the current assembly, ARS1, is discussed elsewhere (14). The goat chromosome 18 scaffold of ARS1 is available in GenBank (CM004579). The sheep chromosome 14 scaffolds within the Oar_v4 and the Oar_rambouillet_v1.0 assemblies were acquired from GenBank (NC_019471 and CM008485, respectively). Although we manually annotated the LRC within Oar_v4, the large number of sequence gaps (*n* = 43) across this region and likely misassembled contigs was determined to be less informative than the Oar_rambouillet_v1.0 assembly which we include in this manuscript.

The Children's Hospital Oakland Research Institute (CHORI)-243 BAC library (https://bacpacresources.org/) is derived from a Texel ram, and used in part to generate the sheep genome reference assembly, Oar_v4 (27). BAC end-sequences for this library were screened *in silico* for sequence similarity to the *KIR* region. Two BAC clones were determined to overlap the sheep *KIR* region: CH243-263M01 and CH243-422J05. LRC sequence data derived from these clones, as well as other closely related assemblies were then used to identify more BAC clones over the sheep *KIR* region, but none were identified. These clones were expanded overnight and DNA was purified using the Qiagen Large Construct Kit and manufacturer's protocol. The DNA was then sequenced using the Pacific Biosciences RSII platform at GATC Biotech (Konstanz, Germany). Read filtering and assembly were conducted using the Pacific Biosciences single molecule real-time (SMRT) analysis software (version 2.3.0; http://www.pacb.com/devnet/). The cloning vector sequence (GenBank: AY487253) was identified and removed, resulting in consensus sequences of ~223 and 172 kb for CH243-422J05 and CH243-263M01, respectively. Alignment of these insert sequences generated an overlap of ~147 kb which contained no differences between the two clones, so they almost certainly belong to the same haplotype. Together, the two assembled clones form a contiguous sequence of 248,501 bp. This assembly was annotated and deposited in GenBank (MH680527).

### Identification and Annotation of LRC Genes

Genomic scaffolds and BAC clones were queried for their gene content using a combination of the basic local alignment search tool (BLAST) (28), the BLAST-like alignment tool (BLAT) (29), HMMgene (30), and NCBI conserved domain search (31). Transmembrane (TM) domains were predicted using TMHMM (32). Identified genes and gene fragments were then manually annotated using Artemis 17.0.1 (33). Exonic structure for the *KIR* genes was determined based on alignment to the previously described cattle *KIR* gene sequences (11). These were visually verified to confirm accuracy, and splice junction donor acceptor sites were identified based on the GT/AG motif (34). Translation of the putative coding region sequence (CDS) enabled prediction of functionally intact genes by identifying frameshifts and nonsense codons.

Genes encoding inhibitory receptors were identified in part based on their potential to produce a protein containing immunoreceptor tyrosine-based inhibitory motifs (ITIMs), which have a loosely conserved amino acid sequence of (hydrophobic)-x-Y-x-x-(hydrophobic) in their intracellular tail, where the hydrophobic residues are typically either valine, leucine, or isoleucine, although serine may occasionally be found at the N-terminal position (35). In contrast, genes for activating receptors were identified based on their possession of a short intracellular tail and a charged residue, such as arginine or lysine, in the predicted TM domain that would enable interaction with an activating adapter molecule such as CD3ζ, FcRγ chain, DAP10, or DAP12. Thus, for the purposes of this manuscript, “long-tailed” and “short-tailed” receptors are synonymous with inhibitory and activating receptors, respectively.

### Phylogenetic Analyses and Subgroup Definitions for LILR and KIR

We previously defined two distinct phylogenetic clades (groups 1 and 2) of *LILR* in the pig (1) and we preserve this nomenclature in the present manuscript. Cattle *KIR* gene groups 1 through five were previously defined (11), and these definitions are also used in the present manuscript. Phylogenetic analysis of goat, sheep, and cattle *KIR* genes was used to place individual goat and sheep *KIR* genes into either one of these five groups, or into the novel presently defined groups 6 and 7. *KIR* gene phylogenetic analysis was performed using the exons encoding the leader and the D0 and D1 Ig domains. The D2 Ig domain, TM, and intracellular regions are less variable and more similar to each other even between distantly related *KIR* and do not provide enough resolution for phylogenetic analyses (10). Sequences were aligned using MUSCLE (36). Phylogenetic trees were constructed using the neighbor-joining method, the Tamura-3 parameter model (37), pairwise deletion, and 1,000 bootstrap iterations within MEGA7 (38).

### Transcriptomic Analyses of Goat and Sheep LRC Ig-Like Genes

Recently generated sheep (23) and goat (24, 25) expression atlas data (BioProject accessions: PRJEB19199 and PRJEB23196, respectively) and T cell expression data (BioProject: PRJEB27455) were used to investigate the expression of the LRC genes identified in this study. Data for alveolar macrophages, bone marrow macrophages, CD4+ cells, CD8+ cells, kidney, liver, spleen, and thymus, were downloaded from both datasets. There are currently no available NK cell (i.e., NCR1+) transcriptomes available for the goat or sheep, so this population was not specifically assessed. Reads corresponding to putative LRC Ig-like genes were identified using BLAST+ (39) and an E-value cut-off of 1 ×10^−10^. We assessed the relative expression profiles of identified transcripts by combining detected read counts from each tissue per species. Raw reads per transcript were then normalized using the RUVg method of the RUVSeq R package (40) using the transcriptional read counts of the *SDHA, PPIA, GAPDH, ACTB, RPL13A*, and *YWHAZ* genes in each tissue as controls and by setting the value of “k” to 1. Normalized read count heat maps were generated using the R ggplot2 package.

## Results

### Gene Content and Structure of the Goat LRC

**Figure 1:**
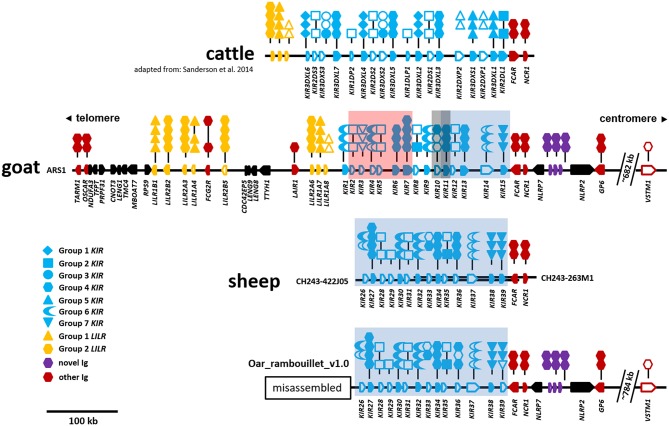
Organization of the LRC in cattle, goats, and sheep. The cattle *KIR* region is shown at top for reference and is adapted from Sanderson et al. (11). Relative gene placement is displayed across a horizontal genomic backbone with centromeric/telomeric orientation indicated at top. Ig-like genes are colored to differentiate between gene groups and gene orientation is indicated with arrows pointing in the direction of transcription. Symbols above each Ig-like gene describe the domain structure with phylogenetic subgroups indicated by different symbols. Long- or short-tailed intracellular domains are shown to indicate whether a gene encodes a putative inhibitory or activating receptor, respectively. Open symbols indicate non-functional domains and pseudogenes, whereas closed symbols indicate that they are putatively functional. The cattle, goat, and sheep (only CH242) assemblies form single unbroken contigs. The separate sheep BAC clones are depicted as parallel overlapping backbones. The Oar_rambouillet_v1.0 sheep assembly (bottom) contains multiple sequence gaps and probably misassemblies telomeric of the region shown. This misassembled region is presented separately in Figure S1. Within the *KIR* region, stretches of sequence that are highly similar between the goat and sheep assemblies are indicated by colored boxes as in **Figure 4** and Figure S1. The location of *VSTM1* is shown as this gene flanks *TARM1* in other species, but is displaced in caprines due to an apparent ancestral chromosomal inversion event.

In the ARS1 assembly, the goat LRC is on the long arm of the telocentric chromosome 18 and ~2.4 Mb from the telomere. The region is flanked by *TARM1* on the telomeric end and *GP6* on the centromeric end, is ~746 kb in size, and contains no sequence gaps (Figure 1). No additional Ig-like genes were identified within 500 kb beyond these genes, suggesting the region is complete. However, the Ig-like gene *VSTM1*, which is adjacent to *TARM1* in pigs and humans, was identified on the opposite end of the LRC and ~682 kb from *GP6*. With the exception of *VSTM1*, the LRC is inverted relative to the centromere compared to other species. These observations are indicatative of a chromosomal inversion with a breakpoint <26 kb downstream (i.e., telomeric in goats) from *TARM1*. The Ig domain of *VSTM1* is also truncated in the goat, possibly rendering that gene non-functional. The gene encoding the Fc receptor for IgA, *FCAR*, which flanks the centromeric end of the *KIR* region, is also inverted in the goat relative to other species, such as cattle, and in the opposite orientation as the *KIR* and *NCR1*. There are a total of 15 *KIR* genes, 8 *LILR* genes, and 3 novel two-domain Ig-like genes in the goat LRC (Figure 1), and of these, seven *KIR*, five *LILR*, and all three novel genes are putatively functional. Detailed information regarding gene structure and genome coordinates for the *LILR, KIR*, and novel Ig-like genes are presented in Table S1.

### Goat LILR Subgroups Are Conserved

**Figure 2:**
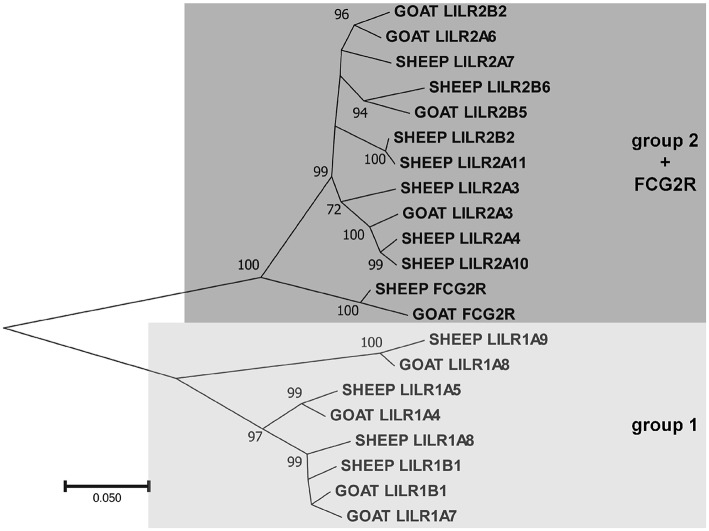
Phylogenetic relationships of goat and sheep *LILR* nucleotide coding region sequences for the extracellular Ig-like domains. *FCG2R* is shown as it is monophyletic with the *LILR*. These two *LILR* groups shown here are based on those previously described in the pig (1). Branch node values indicate the percentage of trees (out of 1,000 iterations) in which the associated sequences cluster together; only values ≥50 are shown. Branch length scale units are number of substitutions per site.

As in other species, the goat *LILR* are present in two distinct clusters separated by the *CDC42EP5*-*LENG9*-*LENG8*-*TTYH1*-*LAIR1* gene cluster (Figure 1). There are two distinct phylogenetic groups of *LILR* in goats and sheep (Figure 2), with two functional group 1 genes (one inhibitory and one activating) and three functional group 2 genes (one inhibitory and two activating). The unique bovid Fc receptor *FCG2R* is encoded in the midst of the *KIR*-distal *LILR* cluster and is phylogenetically most similar to the group 2 *LILR* genes (Figure 2); however, unlike the usual four-domain structure of the *LILR, FCG2R* only encodes an Ig1 domain and an Ig4 domain (Figure 1).

### Novel Goat KIR Genes and Subgroups

**Figure 3:**
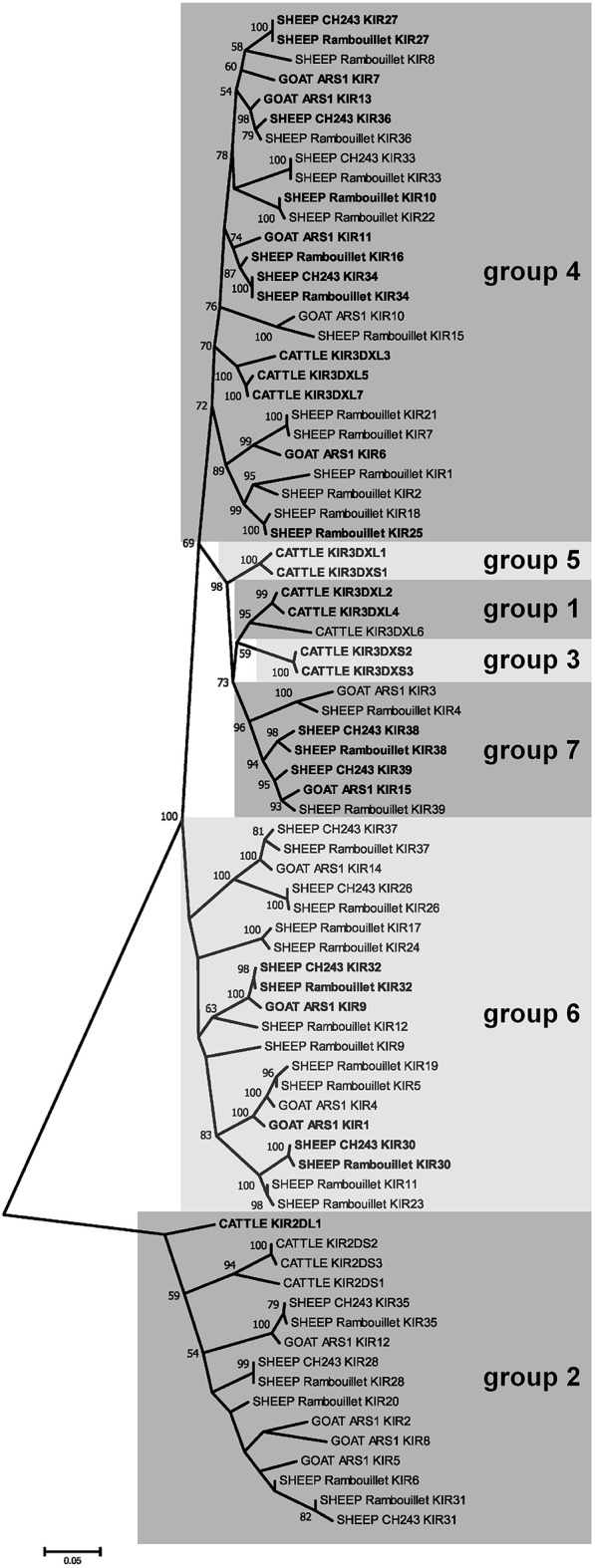
Phylogenetic relationships of goat, sheep, and cattle *KIR* nucleotide coding region sequences for the leader and D0 and D1 Ig-like domains. Four pseudogenes in sheep (*KIR3, KIR13, KIR14*, and *KIR29*) are missing these domains, so they are not shown. Putatively functional genes are shown in bold. Cattle *KIR* sequences and definitions for groups 1–5 were acquired from the previously described cattle haplotype (11). Groups 6 and 7 are novel and so far unique to sheep and goats. Branch node values indicate the percentage of trees (out of 1,000 iterations) in which the associated sequences cluster together; only values ≥50 are shown. Branch length scale units are number of substitutions per site.

Two genes with unusual domain organization were identified. An apparently functional four-domain inhibitory *KIR* gene (*CahiKIR7*) with the domain structure D0-D1-D1-D2, and an inhibitory *KIR* pseudogene (*CahiKIR14*) which contains a ~7 kb retrovirus insertion in the D2 domain. The goat appears to lack a functional group 2 gene, such as *KIR2DL1*, which is present in many other species. Furthermore, the goat lacks genes belonging to phylogenetic groups 1, 3, and 5, which together comprise the majority of the functional *KIR* genes in cattle. Of the previously described clades in cattle, goats only possess functional copies from group 4. In contrast, we identified two novel phylogenetic clades of *KIR* in the goat, which we presently define as groups 6 and 7 (Figure 3). Four *KIR* genes from these novel subgroups appear functional and three are either null alleles on this haplotype or pseudogenes.

### The Structure and Gene Organization of the Sheep and Goat LRC Appears Largely Conserved

Although the sheep genome assemblies are not as complete as the goat, it is possible to observe some common characteristics with the current level of assembly. The sheep LRC is also inverted on the long arm of the telocentric chromosome 14, ~2.9 Mb from the telomere. Despite a recent update and addition of long-read PacBio sequence, the region between *GP6* and *TARM1* within the Oar_v4 (Chr14: 59,516,600–60,255,000) reference assembly is disrupted by 43 sequence gaps and likely incorrectly placed contigs and missing sequence (GenBank: NC_019471). We therefore focused our analyses on the Oar_ramboullet_v1.0 assembly which contains only five sequence gaps across the same region (Figure S1). Within this assembly, the *LILR* are contained on a single contig and are similarly organized as in the goat, although sheep are missing an inhibitory group 2 pseudogene and have an additional activating group 2 gene. Peculiarly, there are two *LILR* genes telomeric of *TARM1* and several gene fragments on an adjacent contig (Figure S1). As far as we are aware, this unusual *LILR* placement is not described in other species, nor is it found in Oar_v4, suggesting that this arrangement is most likely the result of misassembly. Overall, those regions that are well-assembled confirm the goat assembly and provide some evidence of species-specific diversification.

### The Sheep Genome Assembly Contains Two Distinct KIR Haplotypes

**Figure 4:**
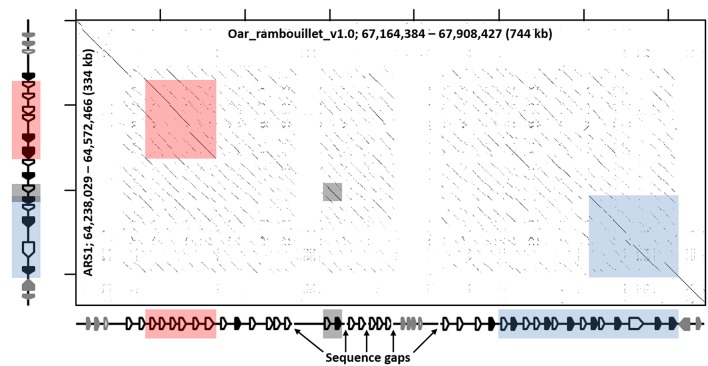
Recurrence plot of goat (*x-axis*) and sheep (*y-axis*) *KIR* regions. The displayed regions span the interval between *LAIR1* and *NLRP7*, exclusive. Gene annotations for the respective assemblies are shown next to the axes, which in the case of sheep is considered putative and likely inaccurate, but represents the current assembly. *Black-shaded* genes represent the *KIR*, and the *gray-shaded* genes represent non-*KIR* Ig-like genes. The shaded boxes indicate regions of high similarity as in Figure 1 and Figure S1.

Manual annotation of the Oar_ramboullet_v1.0 assembly revealed that all five sequence gaps are within the *KIR* region and these gaps separate contigs which contain either *KIR* genes or (presumably) incorrectly placed novel Ig-like genes. There are 39 *KIR* genes and gene fragments within this assembly. These genes are mostly assembled onto two large contigs at the telomeric (14 genes) and centromeric (18 genes) ends of the *KIR* region and seven additional *KIR* genes are assembled to three much smaller contigs (Figure 4, Figure S1). A four-domain *KIR* gene is found on both the telomeric and centromeric contigs, although it is uncertain if this is due to the incorrect assembly of different haplotypes or if it is a real duplication. Phylogenetic analysis (Figure 3) identified at least six pairs of genes that appear to be duplicated across contigs (*ovarKIR17*/*ovarKIR24, ovarKIR18*/*ovarKIR25, ovarKIR19*/*ovarKIR5, ovarKIR21*/*ovarKIR7, ovarKIR22*/*ovarKIR10*, and *ovarKIR23*/*ovarKIR11*). Overall these observations strongly suggest that two distinct and incorrectly phased haplotypes have been at least partially assembled in Oar_ramboullet_v1.0, and that the sequence gaps within the *KIR* region are the result of the impossible assembly of two distinct haplotypes into one reference sequence.

### BAC Assembly of the Sheep KIR Region

To further investigate the sheep *KIR* region, we identified and sequenced two overlapping BAC clones derived from a Texel ram. The 248.5 kb BAC assembly contains 14 *KIR* genes which, although derived from a different breed, match the centromeric *KIR* found in the Oar_ramboullet_v1.0 assembly, thus confirming that haplotype structure (Figure 1). Seven of these *KIR* genes are putatively functional and only one of these is activating (*OvarKIR38*). Although the overall structure is the same, the genes are generally polymorphic between the BAC assembly and the Oar_ramboullet_v1.0 assembly. Of these polymorphisms, two putative indels that could affect gene function are present in *OvarKIR36* and *OvarKIR39* in the Oar_ramboullet_v1.0 but not the BAC assembly. These are likely the result of sequencing errors, of which indels are common in long-read sequencing data.

### Transcriptome Analysis Reveals Unusual Expression of Group 7 KIR

**Figure 5:**
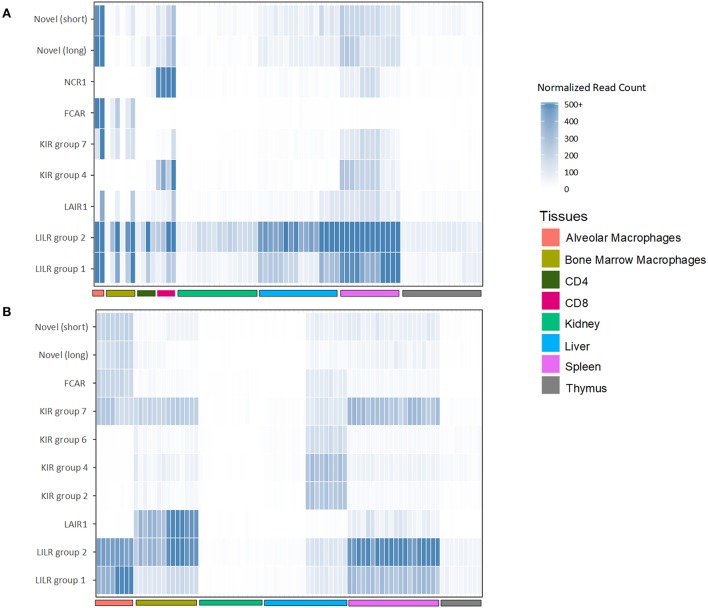
Normalized expression of goat **(A)** and sheep **(B)** LRC genes in selected tissues. The category “*LILR group 2*” includes *FCG2R*. Genes which had <5 normalized reads across all tissues are excluded from the figure. Raw and normalized read counts are shown in Table S2.

To assess the transcription of goat and sheep LRC genes, we queried the sheep and goat gene expression atlases (BioProjects: PRJEB19199 and PRJEB23196, respectively) and goat CD4 and CD8 T cell expression data (BioProject: PRJEB27455). We then normalized the expression based on the read counts for endogenous reference genes (Figure 5, Table S2). Given that these data are based on short reads, which can make distinguishing between highly similar genes problematic, we focused our analyses on the gene subgroups for *KIR* and *LILR*, and on the inhibitory or activating designations for the novel two-domain genes. This revealed that the tissue distribution of LRC gene expression was broadly similar between goats and sheep and largely consistent with leukocyte-associated receptors (Figure 5, Table S2). The *LILR* and the Fc receptor-encoding gene, *FCAR*, for example, were consistently and expectedly expressed by macrophage populations, as were the novel Ig-like genes. Unexpectedly however, the novel *FCAR*-proximal group 7 *KIR* genes were highly expressed in the macrophage populations, whereas the other *KIR* genes were not. While the expression of *NCR1* in all sheep tissues was minimal, goat spleen and CD8 samples were relatively high in *NCR1* expression. However, *NCR1* was not expressed in the macrophage pools, suggesting minimal NK cell contamination in the macrophage preparations (Figure 5). In contrast the *KIR* subgroups were approximately equally expressed in the spleen, while subgroup 4 *KIR* were more highly expressed in the CD8 samples. Additionally, novel Ig-like gene expression, while highest in alveolar macrophages, was higher in CD8 cells compared to CD4 cells. As far as our analyses could determine, the transcriptomic data further confirmed the annotations and gene models that our *in silico* analyses had predicted.

## Discussion

The gross organization of the goat and sheep LRC is typical of other described species, albeit with variation in the number of genes within particular gene groups, such as the *KIR, LILR*, and novel Ig-like receptors. The *KIR* were found to be as expanded as in cattle (11), but with major differences in subgroup phylogeny, indicating that cattle *KIR* and caprine *KIR* have distinct evolutionary histories with independent expansions and/or contractions. Particular *KIR* subgroups consequently have very little shared homology. Of note, goats and sheep lack a functional group 2 (i.e., 3DL-lineage) gene, such as *BotaKIR2DL1*. This gene lineage is found in a wide variety of other species, such as cattle, pigs, horses, cats, seals, and sea lions (7), and is most closely related to the expanded *KIR* genes found in primates (10). The existence of a four-domain *KIR* gene in goats and sheep is also unusual, although four-domain *KIR* genes have also been described in the New World night monkey *Aotus vociferans* (41).

The novel group 7 *KIR* genes proximal to *FCAR* were found to be particularly conserved between goats and sheep, albeit absent from cattle. Transcripts for these receptors were found to be expressed at high levels in macrophages in both sheep and goats. Expression of the group 7 *KIR* correlated with high expression of *FCAR* in the macrophage populations (42). In contrast, expression of *NCR1* (i.e., *NKp46*) which is an NK cell marker (43), was absent, indicating that NK cell contamination was minimal. The coincident expression in macrophages of the group 7 *KIR* and adjacent *FCAR* may suggest that these genes are under similar transcriptional control. Further work is needed to determine the ligands for these *KIR*, and whether these *KIR* genes are specifically found on macrophages, or if they are also present on other distinct cell types, such as NK cell subsets.

As we previously reported for the pig (1), the *LILR* also clade into two distinct subgroups in both sheep and goats. The transcript expression of these receptors is also similar to the pig, in that they were relatively highly expressed in leukocyte-rich tissues. The group 1 genes, however, were less expressed, likely due to the fact that there are fewer functional copies. The gene encoding FCG2R is so far unique to bovid species and is nearly indistinguishable from the group 2 *LILR* genes, particularly in short read sequencing data, and as such we could not differentiate between them.

The novel two-domain Ig-like genes that we previously described in the pig are present as three copies in both the sheep and goat genome assemblies. These genes vary considerably in content between species and can differ between haplotypes (1). Indeed, a misplaced contig in the midst of the *KIR* region in the Oar_ramboullet_v1.0 assembly suggests that sheep haplotypes may also vary in content. Similar to the observed high expression in porcine peripheral blood (1), we presently found these genes to be highly expressed in goat and sheep tissues. This expression seemed to mirror that of the *LILR*, suggesting expression by similar cell types. The function of these novel Ig-like genes remains unknown, but they are widely distributed and conserved across mammalian species, suggesting a conserved function.

Our analyses identified a large number of non-functional *KIR* pseudogenes or null alleles in both the goat and sheep assemblies and a large majority of these have the potential to encode activating receptors. This observation is consistent with the cattle *KIR* (11), human *KIR*, mouse *KLRA* (44), *KLRC* in bovids and horses (9), and *LILR* in pigs (1). Even in humans, relatively little is known about the ligand binding of activating KIR as they tend to have poor affinity for MHC class I ligands compared to the inhibitory KIR. It has recently been shown, however, that the affinity of human KIR2DS1 for HLA-C is greatly increased following human cytomegalovirus infection (45), and that human KIR2DS2 specifically recognizes conserved flaviviral peptides presented by HLA-C^*^0102 (46). The expansion, diversification, and eventual loss of activating receptors may therefore be driven in part by the evolutionary arms race between the host and its pathogens. It is hypothesized that once they are no longer beneficial for host survival and reproduction, activating genes are quickly lost as their retention may permit inappropriate cytotoxicity and autoimmunity (44).

The ligands for the presently discussed LRC receptors are currently unknown outside of primates and rodents. However, only recently has a host ligand for human NKp46 been identified (47), and the ligands for several human LILR are also currently unknown (17), thus highlighting a remaining challenge in leukocyte biology. Due to advances in genome sequencing and assembly, our understanding of the repetitive immune-related gene complexes in non-human species is greatly accelerating. These advancements and the knowledge of the receptors that exist and their diversity are fundamental prerequisites to identifying their ligands, and by extension, their function and involvement in immunity.

## Data Availability Statement

The datasets generated for this study can be found in GenBank, MH680527.

## Author Contributions

JH, JS, NS, DB, and TS conceived the experimental design. JS, NS, and DB performed the experiments and the analyses. JS and JH wrote and edited the manuscript. The paper was reviewed and agreed upon by all authors.

### Conflict of Interest

The authors declare that the research was conducted in the absence of any commercial or financial relationships that could be construed as a potential conflict of interest.

## Supplementary Material

Table S1 Gene structure of goat and sheep *KIR*, *LILR*, and novel Ig-like genes.

Table S2 Raw and normalized gene expression values for LRC genes in goat and sheep tissues and cell types.
