# Supplementary figures and images for: The Structure, Evolution, and Gene Expression Within the Caprine Leukocyte Receptor Complex
Source: Front Immunol. 2019 Sep 26;10:2302. doi: 10.3389/fimmu.2019.02302 (PMC6775213; doi:10.3389/fimmu.2019.02302)

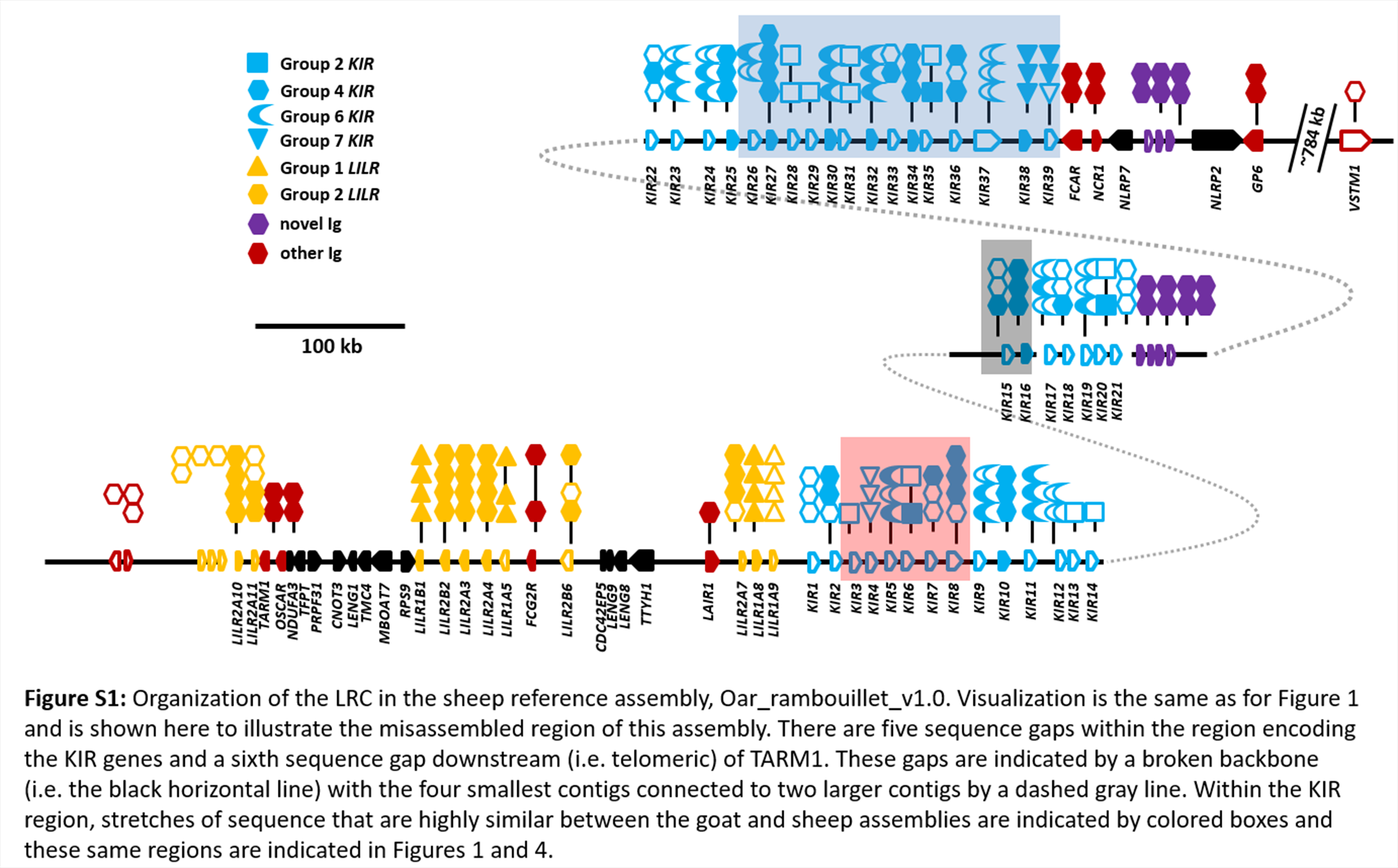

Supplement: Supplementary file 1 [file Image_1.TIF]
